# Improvement in insulin sensitivity and prevention of high fat diet-induced liver pathology using a CXCR2 antagonist

**DOI:** 10.1186/s12933-022-01564-y

**Published:** 2022-07-12

**Authors:** Brett E. Phillips, Louise Lantier, Carl Engman, Yesica Garciafigueroa, Aatur Singhi, Massimo Trucco, Christos Mantzoros, David Wasserman, Nick Giannoukakis

**Affiliations:** 1 Institute of Cellular Therapeutics, Allegheny Health Network, 11th Floor South Tower, 320 East North Avenue, Pittsburgh, PA S15212 USA; 2 Department of Molecular Physiology and Biophysics, Vanderbilt University., Nashville, TN 37232 USA; 3 Department of Pathology, School of Medicine, Room A616.2, UPMC Presbyterian, University of Pittsburgh, 200 Lothrop Street, Pittsburgh, PA 15213 USA; 4 Section of Endocrinology, VA Boston Healthcare System, Harvard Medical School, Boston, USA; 5 Department of Medicine Beth Israel Deaconess Medical Center, Harvard Medical School, Boston, MA USA

**Keywords:** Type 2 diabetes, NAFLD, NASH, Neutrophils, AZD5069

## Abstract

**Background:**

Liver pathology (LP) characteristic of non-alcoholic fatty acid disease (NAFLD)/non-alcoholic steatohepatitis (NASH) is a prevalent co-morbidity of type 2 diabetes (T2D). Accumulating evidence indicates that neutrophils driving insulin resistance (IR), including hepatic IR, precipitate T2D-associated NAFLD/NASH. We hypothesized that targeting neutrophil accumulation into insulin-sensitive tissues in mice using a CXCR2 antagonist under T2D-precipitating high fat diet (HFD) could improve insulin sensitivity and prevent the progression towards liver pathology reminiscent of NAFLD/NASH.

**Methods:**

Mice were age-matched and on standard rodent chow prior to 1:1 randomization into control and HFD formulated with the CXCR2 antagonist AZD5069 or with biologically inactive substitute. They were monitored for metabolic changes including insulin sensitivity using the hyperinsulinemic-euglycemic clamp and hepatic histopathologic evaluation in H&E-stained sections as well as via immunofluorescence microscopy of liver sections for leukocyte markers, collagen 1A1 formation, α-smooth muscle actin (SMA), and galectin-3 expression, for 16 weeks. Statistical tests used to determine significant differences among study groups and outcomes include Student’s t-test, one-way ANOVA, repeated measures two-way ANOVA, and Fisher’s exact test, depending on the analytical question.

**Results:**

Compared to mice on HFD, mice in the AZD5069-formulated HFD exhibited improved insulin sensitivity, a modest reduction in weight gain, and a significant improvement in LP and markers related to NAFLD/NASH. Mice in the AZD5069-formulated HFD also exhibited reduced neutrophil accumulation into the liver at the end of the 16 week study period.

**Conclusions:**

These results show, for the first time, the effectiveness of a selective CXCR2 antagonist to improve insulin sensitivity, concomitantly preventing the progression towards LP characteristic of NAFLD/NASH. This represents a novel approach to target IR and developing LP under T2D-susceptible conditions using a single agent. Furthermore, our data extend the growing evidence in support of neutrophils as a leukocyte population that imprints and maintains a chronic inflammatory state in the progression of dysregulated metabolism in liver-specific co-morbid conditions.

**Supplementary Information:**

The online version contains supplementary material available at 10.1186/s12933-022-01564-y.

## Background

The metabolic syndrome that may include insulin resistance, NAFLD, chronic low-level inflammation, as well as other serious co-morbidities [1]. Precipitated mainly by obesity, the metabolic syndrome may eventually lead to type 2 diabetes (T2D) [1]. Evidence indicate that a number of co-morbid conditions begin to develop even before the onset of impaired glucose tolerance and insulin insensitivity [2]. One of the co-morbidities that is on the rise globally, in terms of prevalence and incidence, is non-alcoholic fatty liver disease (NAFLD) [3]. The liver of healthy subjects maintains steady state circulating glucose that quickly responds to dynamic fluctuations of glucose resulting from feeding, fasting, and physical activity. Obesity precipitates glucose intolerance as well as insulin resistance (IR). The theory is that metabolite accumulation resulting from excess nutrient supply are sensed causing a pro-inflammatory response, especially the innate arm. This results in a hepatic inflammatory state that aggravates an already-precarious state of metabolic control [4]. A number of molecular mechanisms have been identified in the systemic and hepatic inflammatory responses including elevated concentrations of pro-inflammatory immunokines [4], non-physiologic circulating levels of adipokines [5], and hepatic accumulation of leukocytes which together with activated Kupffer cells, or independently of them [6], create a pathologic state of hepatocyte function that contribute to glucose intolerance and IR.

One of the first observations that implicated inflammation in the progression of obesity to the metabolic syndrome and T2D, was the accumulation of activated macrophages inside adipose tissue of obese non-diabetic as well as obese diabetic patients [7]. These macrophages produce a wide array of pro-inflammatory immunokines that confer IR and impaired glucose uptake [8]. While macrophages are not the only leukocytes that progressively-accumulate inside hypertrophic adipose, they are the first—together with neutrophils [9]. Neutrophils comprise the largest population of circulating leukocytes in humans (between 50 and 70% of all circulating leukocytes and together with tissue-resident macrophages are the earliest to be activated and recruited inside an anatomical site that exhibits “damage” and “danger”, including microanatomical regions of hypertrophied adipose, alone or as part of a specific tissue (e.g. skeletal muscle and liver). Activated neutrophils can mobilize tissue-resident leukocytes by producing immunokines, chemokines, as well as by direct contact [10]. Accumulating data indicate that neutrophils are critical leukocytes in the onset of IR and T2D [11, 12].

Systemic neutrophil depletion improves fasting blood glucose levels and prevents high fat diet-induced hepatic structural disorders [13]. Neutrophils are recruited to a site of “damage”/”danger” mainly by the balance of CXCR2 and CXCR4 ligands and the neutrophil cell surface ratio of CXCR2:CXCR4 chemokine receptors [14]. Mice lacking CXCR2 exhibit impaired neutrophil recruitment and reduced tissue damage in a model of acute pancreatitis suggesting a failure to initiate local inflammation processes [15]. CXCR2-deficient mice are protected from high fat diet-induced IR and T2D and are characterized by reduced macrophage accumulation in adipose [16]. One of CXCR2 chemokine ligands, CXCL5, has been shown to be elevated in obese mice and humans, and in mice it was shown to induce IR in a CXCR2-dependent manner [17]. Another CXCR2 ligand, IL-8, was shown to be elevated in the circulation of obese individuals [18] and its steady-state circulating levels correlate well with adiposity and insulin resistance [19].

CXCR2 blockade has been explored as a possible treatment of several neutrophil-driven pathologies [20]. We surmised that impairing neutrophil accumulation could improve insulin sensitivity, prevent the progression of high fat diet induced diabetes, and prevent the progression towards NAFLD. We used a selective CXCR2 antagonist, AZD5069 [20] to test our hypothesis. AZD5069 was initially developed to inhibit neutrophil recruitment into, and activation inside, inflamed airway tissue [20]. Although pre-clinical studies indicated good efficacy in a number of airway inflammatory conditions, outcomes in human trials were not as impressive, even though the study agent was very well-tolerated with few side effects [21]. CXCR2 ligands are expressed in the pancreas, adipose and liver [22], suggesting that under potentially-stressful states, their secretion can be expected to recruit and activate neutrophils, which in turn would exacerbate and amplify a low grade inflammatory condition [23]. In spite of the evidence linking neutrophils and CXCR2 to T2D and possibly T2D-associated NAFLD [17], to date there are no studies aimed at short-circuiting neutrophil function and neutrophil-driven inflammation independently of macrophages—well known participants in the progression of HFD pathology towards NAFLD/NASH [24–35]—in progressive obesity-driven metabolic dysregulation that results in T2D and associated NAFLD. Herein, we show for the first time, using the selective CXCR2 antagonist AZD5069 in high fat fed mice, improved insulin-induced suppression of hepatic glucose production, decreased hepatic lipid storage, and a significant prevention of progression towards liver pathology reminiscent of NAFLD.

## Methods

### Ethics

All animal experiments were approved and conducted in accordance with the AALAC-certified Allegheny Health Network IACUC policies and standard operating procedures as well the AALAC-accredited Vanderbilt University Animal Care and Use Committee policies.

### Animals and diets

C57BL/6 J male mice were purchased from Jackson Laboratory (Bar Harbor) and were maintained under pathogen-free conditions while fed ad libitum a standard chow consisting of 13% kcal fat (control diet; CND; LabDiet, St. Louis). At 6 weeks of age, the mice were randomly assorted into three diet groups: (a) CND; (b) a high fat diet (HFD) consisting of 60% kcal fat (D12492i, Research Diets, New Brunswick); and (c) HFD formulated with AZD5069 (HFD + AZD) at a concentration of 593.8 mg/4057 kcal. AZD5069 was generously provided in-kind by Astra Zeneca and supplied to Resaerch Diets for formulation into HF rodent chow. Mice were maintained on these diets for up to 16 weeks. They were euthanized at pre-defined time points and organs/tissues were collected. Organs were weighed and their dimensions recorded upon resection.

### Control of blood glucose control and insulin sensitivity

Blood was collected at baseline (following randomization into each of the three diets) and then at every 4 weeks thereafter until euthanasia. Mice were fasted overnight (approximately 8 h) prior to blood collection. Blood was obtained following scoring the tip of the tail to measure blood glucose (One Touch Ultra Electronic Glucometer; Lifescan Technologies). Animals then received an intraperitoneal bolus glucose injection (IPGTT; 20% sterile glucose solution, 10 μl per gram body weight) in order to assess glucose tolerance [36]. Tail vein blood was subsequently sampled at 15, 30, 60, and 120 min after the glucose bolus injection. Plasma from each sample was used to measure C-Peptide by ELISA (Crystal Chem catalog #90,050). The area under the curve (AUC) for glucose and C-peptide concentrations were measured to determine glucose tolerance. In addition, to obtain insights into the effects of AZD on insulin sensitivity, we conducted hyperinsulinemic-euglycemic clamps that included radiotracer flux measurements [37]. These were performed at the Vanderbilt Mouse Metabolic Phenotyping Center as previously-described [38]. Baseline blood or plasma variables were calculated as the mean of values obtained in blood samples collected at −15 and −5 min. At time zero, insulin infusion (4 mU/kg body weight/min) was started and continued for 155 min. Blood was sampled from 80 to 120 min for the determination of [3-^3^H]-glucose. Clamp insulin was determined at t = 100 and t = 120 min. At 120 min, 13 µCi of 2-^14^C-deoxyglucose (^14^C-2DG) was administered as an intravenous bolus. Blood was taken from 2 to 25 min for determination of plasma ^14^C-2DG. After the last sample, mice were anesthetized and tissues were frozen for further analysis. Plasma insulin was determined by RIA. Radioactivity of 3-^3^H-glucose and ^14^C-2DG in plasma samples, and ^14^C-2DG-6-phosphate in tissue samples were determined by double label liquid scintillation counting. Glucose appearance (Ra) and disappearance (Rd) rates were determined using steady-state equations. Endogenous glucose appearance (endoRa) was determined by subtracting the total Ra from the glucose infusion rate (GIR). The tissue-specific glucose metabolic index (Rg) was calculated as previously described [39].

### Metabolite, insulin, C-peptide, and adipokine measurements

Commercially-available colorimetric assay kits were used to measure the activity and/or concentration in whole blood or blood plasma of each of the following: Hemoglobin A1c (Catalog #80,310, Crystal Chem, Elk Grove Village); alanine aminotransferase (ALT) activity (Cat#MAK052, MilliporeSigma, St. Louis);, aspartate aminotransferase (AST) activity (Cat#MAK055, MilliporeSigma), triglycerides (Cat#100,103,003, Cayman Chemical), and urea concentration (Cat#MAK006). Plasma adiponectin (Cat#80,569), C-Peptide (Cat#90,050), insulin (Cat#90,080), and leptin (Cat#90,030) concentrations were assessed by ELISA (Crystal Chem). All samples were measured in triplicate and samples from a minimum of three mice were measured per study timepoint.

### Organ/tissue histology and immunofluorescence microscopy

Following euthanasia, livers were excised and placed inside 2.5% paraformaldehyde (Sigma-Aldrich, MO) for 3–4 h. Pancreata were transferred to 30% sucrose (Sigma-Aldrich, MO) overnight and then embedded in NEG-50 (Fisher Chemicals, NJ). 5–10 micron frozen serial sections were subsequently cut. The sections were permeabilized and blocked with 20% Normal Goat Serum for 1 h at room temperature prior to staining with fluorochrome-conjugated antibodies (single or in pairs). The following antibody clones were used: rabbit anti-collagen I (Abcam, ab279711, EPR24331-53), rat anti-galectin-3 (LS Bio, Seattle, Cat# LS-C62617, M3/38), alpha smooth muscle actin (α-SMA; Abcam ab7817, clone 1A4), rabbit anti-IL-18 (Abcam, Cat#ab242022, EPR22249-212), and rat anti-Ly6G (Abcam, ab25377,1:100, clone RB6-8C5). Immunofluorescence images were obtained using a Zeiss AxioObserver Z1 inverted fluorescence microscope with the Zen 2012 Blue Edition software. The number of cells positive for Ly6G and DAPI nuclear stain were determined using QuPath Software version 0.1.2 [40]. IL-18, collagen I, SMA, and galectin-3 content was determined by total fluorescent intensity using MetaMorph imaging software version 7.8.0.0 (MetaMorph, Nashville). Averages were determined from five to nine slides per animal (n = 5–12) per target and depicted as the number of positive cells per field or signal intensity per field for contiguous markers. Values were normalized on a per microscopic field instead of a per cell number as the number of cells per field were significantly different between diets. Additional slides were H&E-stained for NAS and fibrosis scoring by two independent liver pathologists, expert in NASH/NAFLD, and blinded to the animal treatment. NAS scores are the sum of steatosis (range 0–3), lobular inflammation (range 0–3), and hepatocyte ballooning (range 0–2).

### Statistical analysis

Prism 7.0c software (Graphpad, San Diego) was used to analyze the data to determine statistically significant differences amongst treatment groups. Animal outcome data are presented as means ± SD. Laboratory data are presented as means ± SEM. Statistical tests include Student’s t-Test, one-way ANOVA, repeated measures Two-way ANOVA, and Fisher’s exact test, depending on the analytical question. Post-tests include Tukey’s multiple comparisons and Cochran-Armitage test for trend analysis. In all statistical analyses, a p value of < 0.05 between or amongst treatment groups (actual or Bonferroni-corrected for greater than 2 variables) was considered statistically significant.

## Results

### Impact of AZD5069 on weight, glucose control, and circulating metabolites

**Fig. 1:**
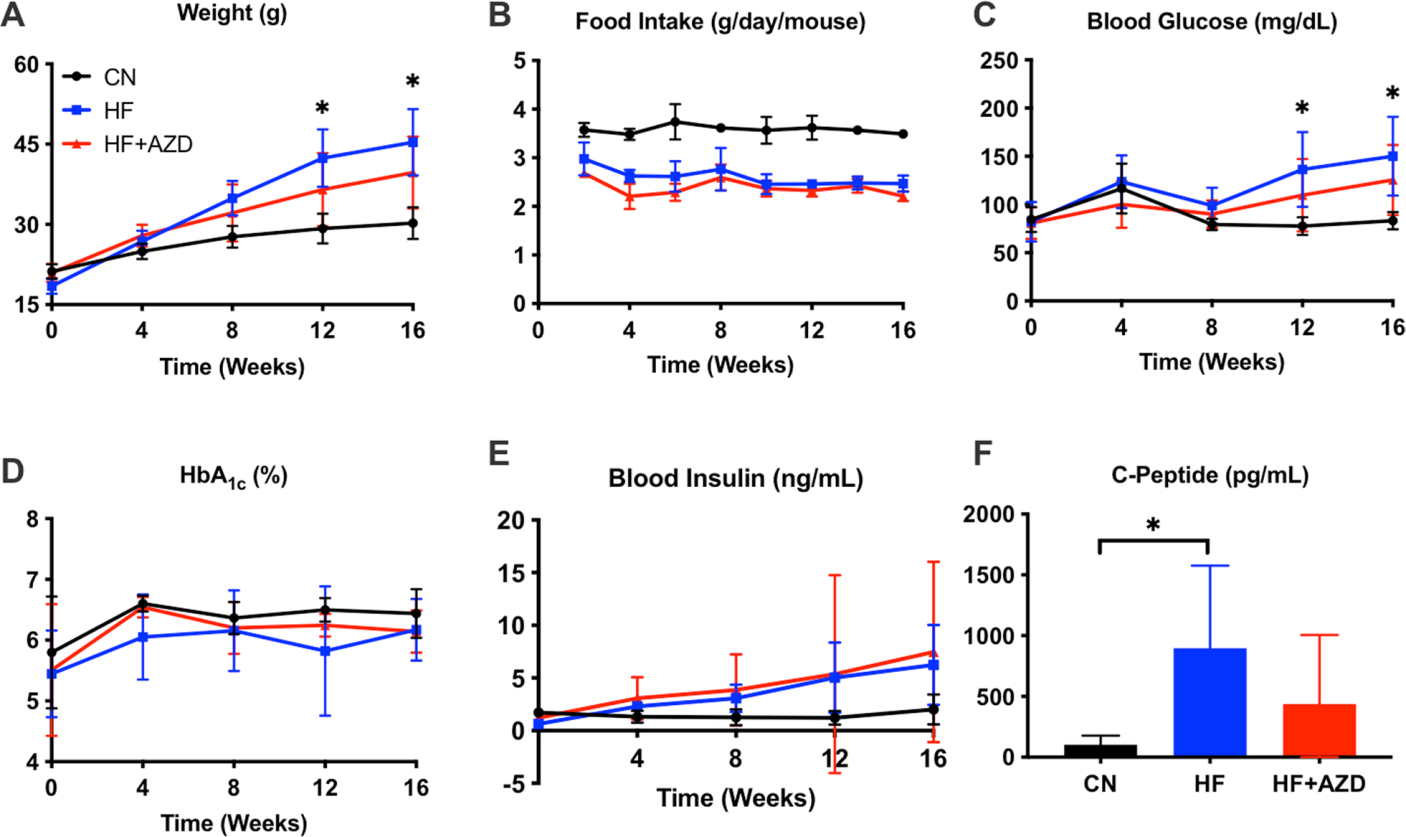
Mice on AZD5069-formulated high fat diet exhibit a small trend towards improvement in weight, fasting blood glucose, and C-Peptide. **A** Animal weight begins to diverge at 8 weeks on diets and is significantly different among animals in all study arms at 12 and 16 weeks. **B** Biweekly food intake in mice in the CN arm was significantly higher compared to mice in the other two study arms, however, weight differences in mice between the HF and HF + AZD diet arms were not due to differences in food intake. **C** Fasting blood glucose concentrations are significantly different in mice between CN and the two HF diet arms at 12 and 16 weeks, but were not reduced in mice in the HF + AZD arm compared to HF diet arm. **D** Hemoglobin A1c levels were not statistically-different among the three study arms at any of the measurement time points. **E** Insulin concentration in blood in mice among the three study was not statistically-different, even though the mice in the HF arms exhibited a trend towards elevated insulin. HF + AZD had no effect on insulin concentrations compared to HF diet alone. **F** C-Peptide concentration in blood. Although there was a trend towards a lower concentration in fasting blood C-Peptide at 16 weeks in mice in the HF + AZD arm compared to those in the HF arm, it was not statistically-distinguishable. The symbols show the median of each of the measurements among n = 8 mice in each of the diet arms and the bars show the SD. Statistical analysis was performed with repeated measures two-way ANOVA or a one-way ANOVA with Bonferonni post tests as appropriate. Significant differences in outcomes among the treatment arms are shown with an asterisk (*), where the p value is < 0.05

Animals placed on HFD showed a significant divergence in weight starting at 12 weeks of age compared to chow fed mice. While HFD-treated mice exhibited an increased weight over CN diet-treated animals, HFD + AZD-treated animals exhibited an intermediate weight between the two extremes (Fig. 1A). These differences were not attributable to differences in diet consumption (Fig. 1B). Fasting blood glucose levels were similar at 12 and 16 weeks between the HFD-fed and the HFD + AZD-groups groups and these levels were higher in both groups when compared to the CN diet group. However, there were no significant blood glucose difference in mice consuming HFD or HFD + AZD (Fig. 1C). Other than a significantly-greater C-peptide concentration in the blood of HFD versus CN diet-treated mice at 16 weeks, we did not discern any differences in HbA1c (Fig. 1D), fasting insulin (Fig. 1E), and fasting C-peptide (Fig. 1F) among the different diet treatment groups.

### AZD5069 treatment does not affect glucose tolerance but improves insulin sensitivity in HFD fed mice

**Fig. 2:**
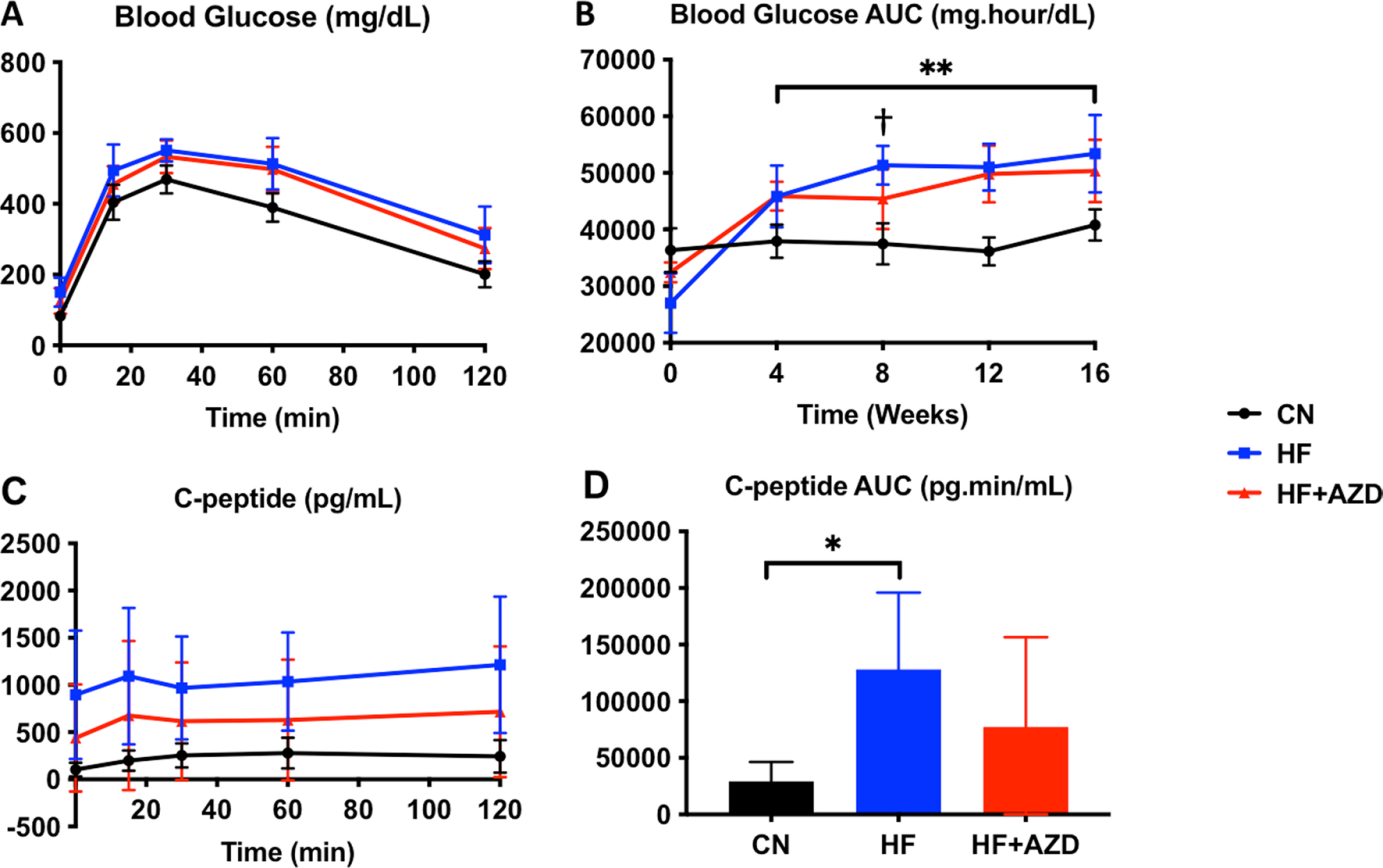
AZD5069 does not improve glucose tolerance under high fat diet conditions. **A** The graph shows the glucose concentration time curve over 2 h following a glucose bolus injection in mice at 16 weeks on each of the treatment arms. **B** IPGTT AUC in mice in the three diet arms over the 16 week study period was significantly higher in mice in the HF and the HF + AZD diet arms compared to CN diet as indicated (**). The only time point where AUC in mice in the HF and HF + AZD diet arms were significantly different from each other was at the 8 week time point (†). **C** C-Peptide concentrations were measured during the same IPGTT experiments. **D** C-Peptide AUC was not significantly different in mice among the three diet arms, although mice on the HF diet exhibited significantly higher C-Peptide AUC than those in the CN diet in the IPGTT at the 16 week time point. The symbols in graphs **A**–**C** and the bars in graph **D** show the median of each of the measurements among n = 8 mice in each of the diet arms and the error bars show the SD. Statistical analysis was performed with repeated measures two-way ANOVA or a one-way ANOVA with Bonferonni post tests as appropriate. Significant differences are shown with an asterisk (*) or a dagger (†) where p < 0.05 and ** where p < 0.01

**Fig. 3:**
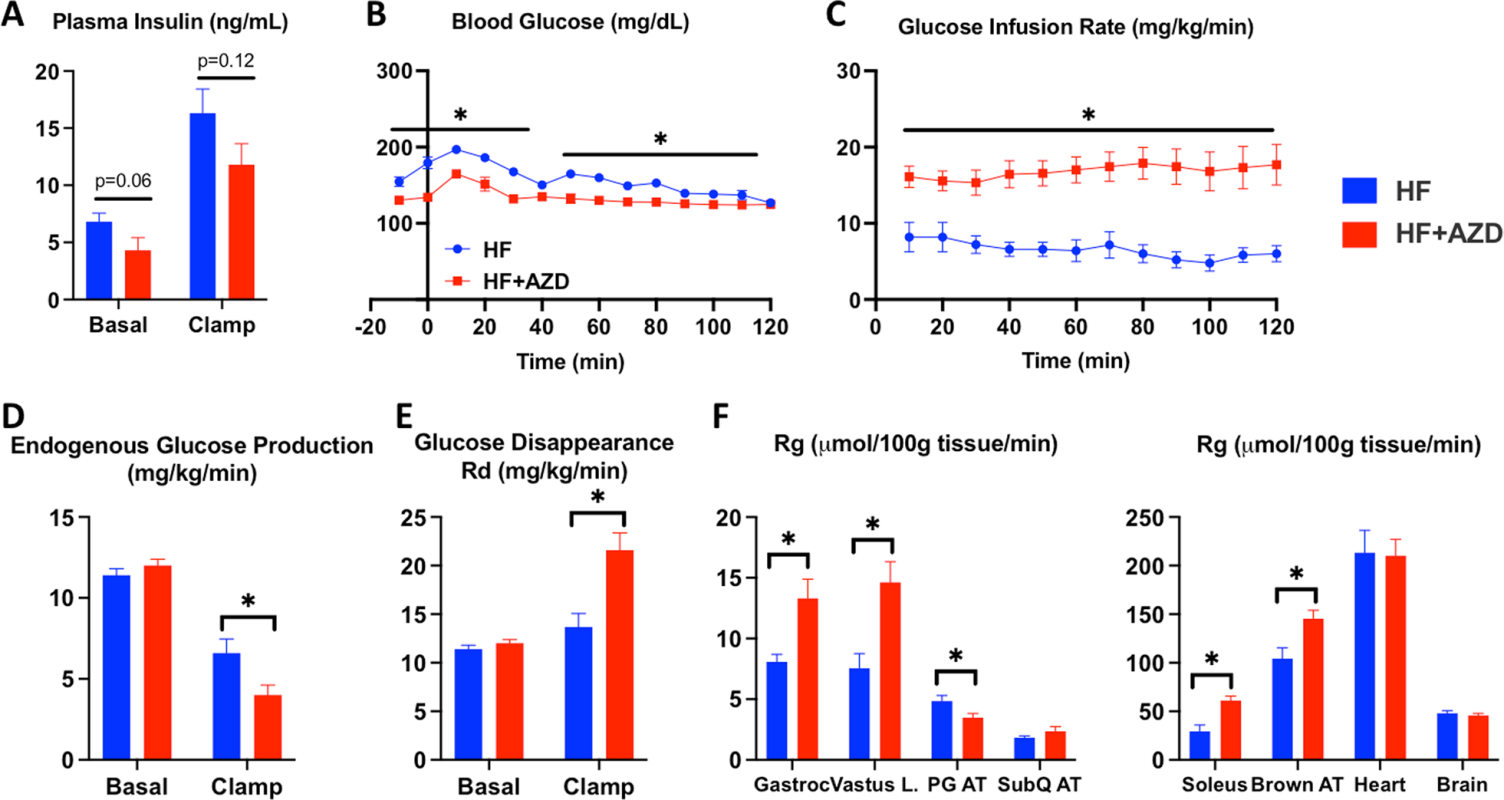
AZD5069 improves insulin sensitivity under high fat diet conditions, as measured by the hyperinsulinemic-euglycermic clamp at 16 weeks. Circulating insulin levels trended lower in mice in the HF + AZD diet arm but were not significantly different. Mouse blood glucose levels during the clamp procedure. The symbols indicate the mean while the error bars indicate the SEM. Glucose infusion rates were significantly higher in mice in the HF + AZD diet arm compared to mice in the HF despite a trend of reduced level of plasma insulin. Endogenous glucose production is unaltered in mice among the diet arms until the clamp is started. Glucose flux (Rd) is unaltered in mice among the diet arms until the clamp is started. Significantly greater 2-DG uptake in skeletal muscle and brown adipose tissue in mice in the HF + AZD arm compared to mice in the other diet arms. PG AT refers to the peri-gonadal white adipose tissue and SubQ AT refers to the resected subcutaneous total fat. The bars in graphs **A**, **D**–**F** and the symbols in graphs **B**, **C** show the median of each of the measurements among n = 6–12 mice in each of the diet arms and the error bars show the SD. Statistical analysis was performed with a Student’s t-test or repeated measures two-way ANOVA as appropriate. Significant differences are depicted with “*” were p < 0.05

Using IPGTT, we identified a significant increase in blood glucose AUC in both the HFD and HFD + AZD diet groups compared to the CN diet group (Fig. 2A, B) as early as 4 weeks post-randomization into each of the diet arms. Outcomes of glucose AUC in the HFD and HFD + AZD groups were only significantly different from each other at 8 weeks of age. C-peptide AUC levels were measured at 16 weeks of age and exhibited significant differences only between the CN diet and HFD (Fig. 2C, D). To determine insulin sensitivity of mice in each of the treatment arms, we conducted the very sensitive hyperinsulinemic-euglycemic clamp [37] in mice in each arm at baseline and after 16 weeks on diet. There was a non-significant trend of increased plasma insulin and glucose in HFD group at baseline compared to those in the HFD + AZD group that persisted throughout the course of the experiment (Fig. 3A, B). Despite having higher insulin levels, HFD-treated animals exhibited significantly-reduced glucose infusion rate (GIR) when compared to the HFD + AZD arm (Fig. 3C). Endogenous glucose production in mice in the HFD + AZD arm was reduced (Fig. 3D) while Rd was significantly elevated under the clamped condition (Fig. 3E). Rg was significantly higher in leg skeletal muscle as well as in brown adipose tissue with HFD + AZD compared to HFD (Fig. 3F). Interestingly perigonadal white adipose tissue exhibited a decline in glucose uptake in the HFD + AZD arm (Fig. 3F). These data support our hypothesis that AZD improves insulin sensitivity in muscle and liver.

### AZD5069 effects on circulating liver metabolites

**Fig. 4:**
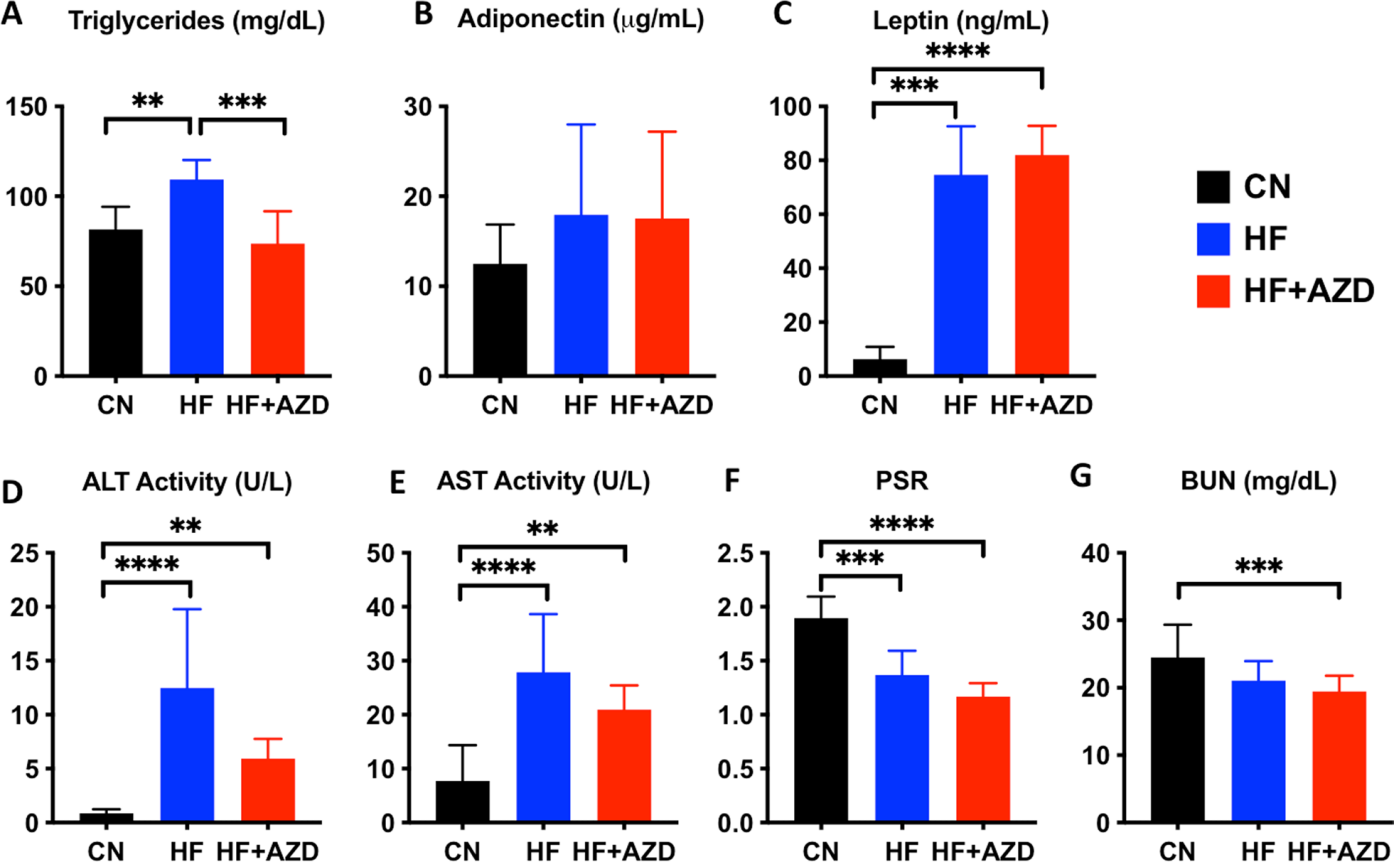
AZD5069 improves the concentration of circulating triglycerides, trends toward improved circulating ALT/AST, and improved platelet number to spleen cross-sectional area ratio (PSR) in mice under 16 weeks of high fat diet. AZD5069 restored circulating plasma triglyceride concentrations those in mice in the CN diet arm (**A**), while circulating adiponectin (**B**) and leptin (**C**) concentrations were not different in mice in the HF and HF + AZD diets. Circulating liver enzymes ALT (**D**) and AST (**E**) in mice in the HF + AZD diet arms trended towards a concentration intermediate between that in mice on the CN and HF diets (p = 0.0541 HF vs. HF + AZD). PSR was reduced in in mice in the HF and HF + AZD diet arms compared to mice in the CN arm (**F**). Liver and Kidney clearance of BUN was significantly reduced in mice in the HF + AZD diet arm compared to mice in the CN diet arm (**G**), but these values were well-within the normal reference range in mice (8.0 to 33.0 mg/dL). The bars in each graph show the median of each of the measurements among n = 6–12 mice in each of the diet arms and the error bars show the SD. Statistical analysis was conducted by one-way ANOVA or Kruskal–Wallis test followed by Bonferroni post-test as appropriate. Significant differences in the medians among the diet arms are depicted with *p < 0.05, **p < 0.01, ***p < 0.001, and ****p < 0.0001

Based on the effects of the study agent on enhanced glucose uptake in muscle and decreased uptake in adipose tissue, its effect on circulating triglycerides levels was subsequently assessed. Mice on the HFD + AZD diet arm exhibited reduced triglycerides, close to the levels in CN diet-treated mice (Fig. 4A). Adiponectin and leptin concentrations, however, were unaltered in mice in the HFD and HFD + AZD diet arms, although leptin was elevated in both HFD groups compared to the CND group (Fig. 4B, C. Plasma ALT and AST were elevated in the HFD while their levels in mice in the HFD + AZD diet arm were intermediate between those in mice in the HFD and CN diet arms (Fig. 4D, E).

A clinical predictor of T2D-associated liver pathology is the platelet-to-spleen ratio (PSR), the ratio of platelet blood concentration to spleen dimensions (length or diameter) [41]. A low PSR is an indicator of heightened liver pathology risk. Due to the small spleen size of mice, we measured spleen cross-sectional area. In Fig. 4F we show that the PSR is significantly-decreased in mice in the HFD as well as the HFD + AZD diet arms compared to the CN diet arm. BUN was significantly-decreased in mice in the HFD + AZD diet arm compared to the CN diet arm (Fig. 4G), however, it was inside the normal range of physiologic BUN, suggesting an adequate clearance of hepatic and renal nitrogen.

### AZD5069 treatment significantly improves liver pathology reminiscent of NAFLD, while reducing lipid content and hepatic neutrophil accumulation

**Fig. 5:**
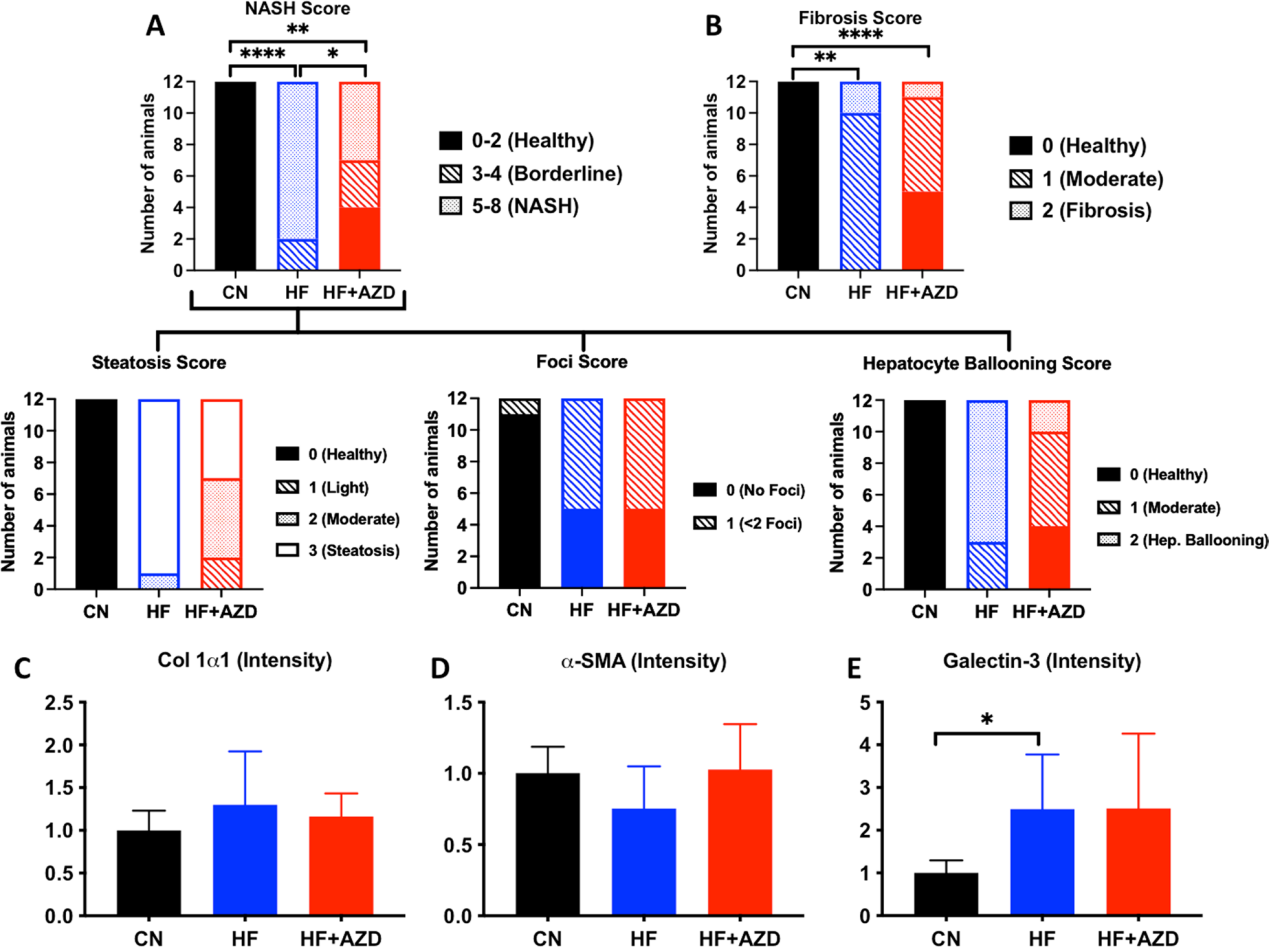
AZD5069 prevents the deterioration of hepatic parenchyma, reduces fibrosis, steatosis, lobulation, and hepatocyte ballooning under high fat diet conditions. The graph shows the median NAFLD Activity Score (NAS) comprised of pathologist-determined scores indicative of steatohepatitis (NASH), inconclusive of steatohepatitis (Borderline) and absence of steatohepatits (Health). H&E liver sections procured from n = 12 mice on 16 weeks of the CN, HF, and HF + AZD diet arms were evaluated for their clinical NAS Score, which consists of steatosis, lobulation, inflammation, and hepatocyte ballooning. A trend in fibrosis improvement was observed in liver sections from mice on HF + AZD at 16 weeks, however it failed to reach statistical significance when compared to assessments of liver sections from mice on HF diet. The graph represents the median of scores assigned by two independent pathologists (n = 12 mice per diet arm). **C**–**E** Positivity for Col1a1, α-SMA, and Galectin-3 in immunofluorescence in serial sections of livers from mice among the three diet arms at 16 weeks (n = 7 mice per diet arm). The bars in each graph show the median of each of the measurements among mice in each of the diet arms and the error bars show the SD. Statistical analysis was performed by Fisher’s Exact test followed by a Cochran-Armitage post-test for trend or the Kruskal–Wallis test followed by Bonferroni post-test. Significant differences depicted with (*) were p < 0.05, (**) were p < 0.01, and (****) were p < 0.0001

We assessed liver histopathology to examine progression of NAFLD in HFD + AZD mice compared to those in the HFD arm. At 16 weeks, H&E-stained liver sections were scored for steatosis, lobular inflammation, and hepatoceullar ballooning following the guidelines of the commonly-accepted composite clinical NAFLD Activity Score (NAS) [42]. As expected, mice on the HFD exhibited worsen composite NAS compared to the mice in the CND treatment arm (Fig. 5A and Additional file 1: Figure S1A). Remarkably, and in contrast, the mice in the HFD + AZD treatment arm exhibited a significantly-decreased composite NAS compared to the HFD arm (Fig. 5A). We noted significantly improved steatosis and hepatocyte ballooning subscores in these sections (Fig. 5A and Additional file 1: Figure S1A). In terms of liver fibrosis, we discerned an improvement in the HFD + AZD treatment arm, however the difference in the score compared to the HFD treatment arm was not significant when Bonferroni correction was applied to adjust for repeated comparisons (p = 0.032 Bonferroni-adjusted vs. target p = 0.016; Fig. 5B). Liver sections were further examined for the early fibrosis markers collagen 1a1, α-SMA, and galectin-3 by immunofluorescence microscopy (Fig. 5C–E and Additional file 1: Figure S1B). The density per unit area of positive fluorescence was unaltered among sections from mice treated with either of the diets, the exception being galectin-3 where the density per unit area in sections from the livers of mice in the HFD and HFD + AZD diets was increased compared to those in the sections from livers of mice in the CN diet arm (Fig. 5C–E and Additional file 1: Figure S1B). Liver weights and cellular density were significantly different between diets (Additional file 2: Figure S2), with the consequence that quantification of immunofluorescence spots and density are noted as fluorescence intensity per field (unit area).

**Fig. 6:**
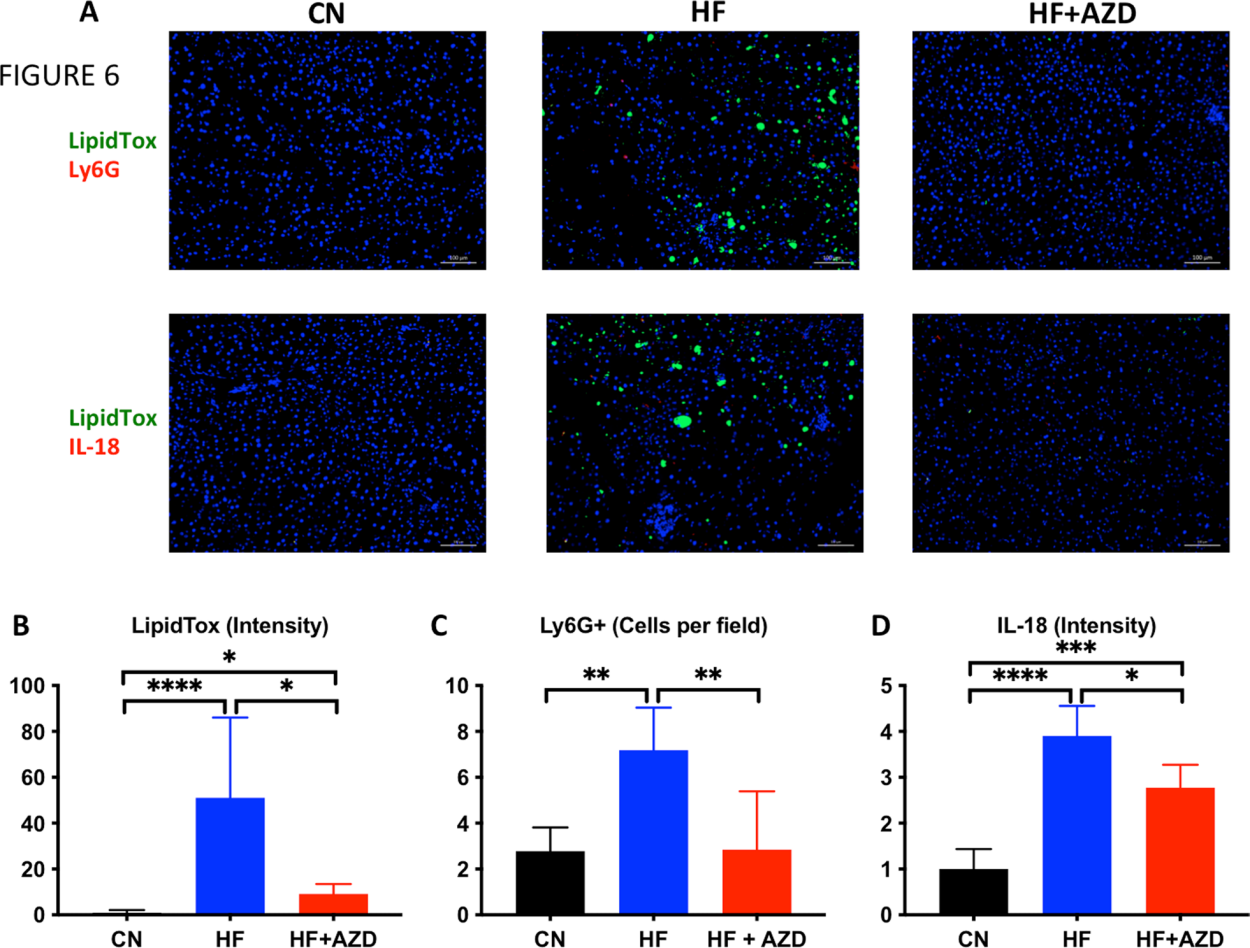
AZD5069, under high fat diet conditions, prevents the accumulation of lipid droplets as well as activated neutrophils in liver. Livers from mice at 16 weeks under each of the study diets were evaluated for the presence of lipid droplets and activated neutrophils by immunofluorescence microscopy. **A** Representative images of livers stained with the lipid indicator LipidTox, the neutrophil marker Ly6G, and the activated inflammasome marker IL-18. **B–D** Quantifications of marker content or cell number per fixed tissue area are shown. HF + AZD displays the accumulation of lipid droplets and IL-18 + cells in livers of mice on the HF + AZD diet is intermediate to those of livers from mice on the CN and HF diets, whereas, the accumulation of Ly6G + cells (neutrophils) is statistically-indistinguishable from that of livers of mice in the CN diet. In graphs **B**–**D**, the bars show the median of the measurements in liver sections of n = 5–9 mice of each diet arm and the error bars show the SD. Significant differences were ascertained by one-way ANOVA or Kruskal-Wallis test followed by Bonferroni post-test. Significant differences are shown where *p < 0.05, **p < 0.01, ***p < 0.001, and ****p < 0.0001

Mice in the HFD + AZD arm exhibited a reduction in hepatic lipid content compared to those in the HFD treatment arm (Fig. 6A, B), approaching densities and patterns close to those seen in liver sections from mice in the CN diet treatment arm, despite the differences in animal weight and liver size. Finally, we observed reductions in the number of accumulated neutrophils (Fig. 6A, C) as well as the density of IL-18 immunofluorescence signals and their intensity, reflective of a reduction in the inflammasome activity in these cells (Fig. 6A, D), in the livers of HFD + AZD mice compared to the HFD arm. These data suggest AZD can effectively limit neutrophil accumulation as well as neutrophil inflammasome activation inside the livers of mice on an obesogenic, IR-inducing HFD that also concomitantly causes a liver pathology reminiscent of NAFLD.

## Discussion

“Pre-diabetes” is characterized by impaired glucose tolerance with an insidious onset of IR, even though fasting plasma glucose (FPG) and HbA1c values may well be within a range considered physiological. If treated early, the metabolic syndrome—mostly reflected as clinical T2D onset—can be delayed or prevented [43]. While metformin is often the first approach, it is neither insufficient on its own to prevent the progression towards T2D nor to attenuate the underlying low grade chronic inflammation which accompanies the progression towards IR in insulin-sensitive tissues as well as the impairment of pancreatic β cell function [44]. Low-grade systemic inflammation persists during clinical T2D [45]. Hyperglycemia and hyperlipidemia, characteristic of T2D, further fuel inflammation, adding to the impairment and damage to insulin-producing and insulin-sensitive tissues and organs through the mechanisms that underlie glucose toxicity and lipotoxicity. By intercepting key immune cellular actors driving this inflammation and/or activating those that attenuate or suppress it, we postulated we could improve insulin sensitivity and β cell function. Improvements in these variables can lead to a decreased risk of developing NAFLD and T2D.

Epidemiologic evidence indicates that at least 50% of individuals diagnosed with T2D also exhibit NAFLD/NASH [46, 47] which, independent of all other T2D comorbid conditions, can accelerate the progression of metabolic and cardiovascular diseases [47]. In obese individuals, NAFLD/NASH is a co-morbidity that coincides with IR [48]. There is strong evidence that inflammation driving IR promotes metabolic abnormalities in liver [49]. Among all leukocyte populations other than macrophages, neutrophils appear to be just as, if not more relevant, in the onset and progression of obesity-driven IR [12, 50–52]. Moreover increasing evidence suggest that neutrophils precipitate NAFLD/NASH in obese T2D patients [53, 54].

Neutrophils are a leukocyte population that, with macrophages [24–35], are the earliest to accumulate in insulin-sensitive tissues with weight gain [12, 50, 51]. We therefore tested whether targeting only neutrophil accumulation independently of macrophages—whose role is already well-established in the progression of NAFLD/NASH [24–35]—could improve insulin sensitivity, prevent the progression of HFD-induced IR, and possibly prevent the progression towards a liver pathology that could be prodromal of, and/or precipitate NAFLD/NASH. Despite a mechanistic rationale linking neutrophils and CXCR2 to IR and possibly IR-associated NAFLD [17], to date, there are no studies aimed at short-circuiting only neutrophil function and only neutrophil-driven inflammation in progressive obesity-driven metabolic dysregulation that results in IR and associated liver pathology precipitating NAFLD/NASH. We chose to use a selective CXCR2 antagonist, AZD5069 [20] since the pancreas, adipose and liver express CXCR2 ligands [22] under stress conditions. Hence, such ligands are able to recruit and activate neutrophils, which in turn may amplify a low grade inflammatory condition [23]. Use of this selective CXCR2 antagonist also allowed us to dissect the effect of neutrophil-selective impairment independently of macrophages on insulin sensitivity and liver pathology under HFD conditions.

The major findings of this study are that AZD5069 formulated HFD that cause causes liver pathology reminiscent of NAFLD with features suggesting NASH, can improve insulin sensitivity, modestly prevent weight gain and reduce fasting glucose in mice fed a HFD. Remarkably, mice treated with AZD5069 exhibited an improvement in liver pathology with reduced lobular inflammation and neutrophil accumulation, decreased hepatocyte ballooning (Additional file 1: Figure S1A) concomitant with improved NAS score. At the mechanistic level, AZD5069 may be beneficial on attenuating the progression of T2D-associated NAFLD/NASH most probably by preventing the activation and minimizing the accumulation of neutrophils into the liver, independently of macrophages, as well as inside the peripheral insulin-sensitive tissues where HFD causes adipose hypertrophy and accumulation. Although our outcomes support a protective role for AZD5060 in the progression of impaired insulin sensitivity and liver pathology, it remains to be determined whether this agent or others in its drug class (CXCR2 antagonists alone) can be effective when treatment is initiated at a later stage of pathology to reverse the progression of hepatic lesions reminiscent of NAFLD/NASH. Other studies in HFD models of NAFLD/NASH have observed improved liver pathology and NAS score under neutrophil-depleting conditions [13]. Additionally, AZD5069 treatment also reduced liver lipid content and hepatic glucose production, both hallmarks of IR in NALFD/NASH. In this study, we measured adiponectin because of its known anti-inflammatory and insulin-sensitizing properties [55]. We hypothesized that AZD5069 would reduce neutrophil accumulation in hypertrophic adipose tissue with the benefit of removing restraint on adiponectin production by neutrophil-produced immunokines. However, our data did not support this hypothesis as an increase in adiponectin was not increased in HFD + AZD fed mice compared to those in the HFD arm (Fig. 4B).

It is worth noting that, although neutrophils are expected to be the primary cells targeted by AZD5069, there are other tissues where the agent may be exerting its actions in its overall effects on improvement of insulin sensitivity. For example, previous studies have shown a CXCR2 mediated inhibitory effect on insulin-induced glucose transport in muscle cells mediated by activation of the JAK/STAT pathway and stimulation of the expression of SOCS-2, a known insulin receptor inhibitor [56]. In addition, scientific evidence highlights IL-8 as a main adipocytokine producing insulin resistance via the inhibition of insulin-induced Akt phosphorylation in adipocytes [57, 58]. Furthermore, very recent data highlighted a specific role of CXCR2 expressed in adipocytes in the modulation of murine adipocyte response to high glucose concentration and shed light on its role in the regulation of the proinflammatory response and insulin sensitivity [59]. Thus, CXCR2 antagonists may very well be acting at multiple sites to achieve a global improvement in insulin sensitivity. This furthers strengthens the rationale that these agents can be potentially helpful treatments in insulin resistance-incident obesity and T2D. We extend these observations and suggest that this class of compounds may also be important, through these same mechanisms, to improve T2D-associated liver pathology such as NAFLD/NASH.

HFD-induced, increased hepatic lipogenesis is an important metabolic abnormality underlying the pathogenesis of hepatic steatosis in insulin-resistant livers. NASH is histologically similar to alcohol-induced steatohepatitis, a disease that can progress to cirrhosis and liver failure. Many of the factors implicated in the development of alcoholic steatohepatitis are also known to be associated with NASH. These factors can be grouped into two broad categories: factors causing an increase in oxidative stress and factors promoting expression of proinflammatory cytokines. Briefly, under HFD conditions, triglyceride and free fatty acid formation contributes to peripheral as well as hepatic insulin resistance [60–65]. The mechanisms in liver, previously-identified, include increased free fatty acid oxidation, oxidative stress and free fatty acid-induced upregulation and activation of PPARγ and AMP Kinase. Together, these diet-induced effects can cause hepatocytes to metabolically-shift towards a pro-apoptotic, inflammation-triggering state. Cui et al. [66] have shown that a CXCL8 analog, referred to as K11R/G31P, designed to be an antagonist of CXCL8 ligands, binding to CXCR1 and CXCR2 receptors, prevented progression of insulin resistance and liver pathology reminiscent of hepatosteatosis, when administered into young *db/db* mice. Along with reduced accumulation of neutrophils into liver, these investigators also discovered that antagonist administration was associated with suppression of gluconeogenesis by decreased hepatic glucose-6-phosphatase and phosphoenol pyruvate carboxykinase activities. Additionally, they observed increased levels of phosphorylated Akt in liver [66]. It remains to be determined if AZD5069, which targets only CXCR2 without any action on macrophages, or other agents in its drug class interfere or modify these biochemical, enzymatic, and biological processes, directly or indirectly, thus strengthing the view that neutrophils are primary leukocytes in driving high fat diet/obesity-associated liver pathology. While CXCR2 antagonism prevents the accumulation of neutrophils inside inflamed liver, its effects on metabolic activity are very likely to contribute, independently of effects on neutrophils, to the effects we observed in this study. The expression of CXCR2 on insulin-responsive muscle and adipose indicates that AZD5069 could be acting on these tissues, concomitant with actions on neutrophils, as CXCR2 activation in muscle and adipose results in impaired insulin-induced glucose transport [56] and insulin resistance [57, 58].

Features of NASH in the progressive liver pathology was observed reliably in our HFD-only treated mice. We are very aware of the limitations of the HFD used in this study in its ability to cause clinically- and molecularly-distinguishable NASH and we acknowledge that other diets can be more “NASHogenic” [67–69]. However, there is an ongoing and vigorous discussion concerning which of these diets can elicit insulin resistance and verifiable NASH concomitantly [67–69]. We therefore chose, in this study, to use a HFD that is generally accepted to concomitantly cause insulin resistance and liver pathology inside a reasonable timeframe. Inflammation and steatosis were consistently seen in liver sections from mice in the control treatment arm. The pathology resembles what is often observed in liver biopsies from humans exhibiting NAFLD/NASH. The most remarkable finding in liver sections from mice treated with AZD5069 was an improved composite NAS score. NAS is a widely-accepted method of evaluating human NAFLD and NASH [70, 71]. Recently, NAS has been shown to be verifiably-associated with change in fibrosis stage in humans [72]. In our study, the lower NAS score in liver sections from mice treated with AZD5069 is a consequence of attenuated lobular inflammation, steatosis, and hepatocyte ballooning. In contrast to the composite NAS score, the attenuation in fibrosis in the liver sections of AZD5069-treated mice was moderate without any signs of regression. Nevertheless, the lower density of collagen 1A1, α-SMA, and galectin-3; molecules that reflect the progression of liver fibrosis and macrophage activation [73], suggests that AZD5069, acting to prevent the activation and accumulation of neutrophils into the liver, can achieve some protective effect against fibrosis. Increased α-SMA and collagen 1a1 levels reflect the activation of hepatic stellate cells and their mobilization into myofibroblast-like cells [74]. Hence, the effect of AZD5069 acting to prevent the activation and accumulation of neutrophils into the liver, may disallow this conversion, thereby preventing collagen deposition and fibrosis. The unraveling of these potential mechanisms, where neutrophils and a neutrophil-based microenvironmental inflammatory state drive them, compel further study of CXCR2 antagonists alone or in combination with other drugs that act at different stages of the pathophysiology of the disease. For example, medications that decrease body weight and thus % fat in the liver, incretins that act by both decreasing liver fat and improving inflammation, other insulin sensitizers such as pioglitazone or CHS-131 that act as PPARγ agonists and finally with medications that may act as antifibrotic agents.

A limitation of our study, herein, are the known differences between human and rodents in regard of CXCR1/2 expression and function. In humans, IL-8/CXCL8 (and CXCL6, also known as GCP-2) exerts its activity by activating both CXCR1 and CXCR2 [75–77], whereas the other ELR-CXC chemokines selectively bind CXCR2 [77]. By contrast, up until recently, only one functional ELR-CXC receptor was identified in mice and was characterized as the homologue of human CXCR2 [78, 79] however, the orthologous murine CXCR1 [80, 81] was identified and subsequently confirmed to be a functional receptor [82], specifically activated by murine GCP-2, human GCP-2/CXCL6, and human IL-8/CXCL8. Thus, given possible differences between mice and humans, the data obtained herein using CXCR2 selective inhibition by AZD5069 caution that further studies in humans are warranted and the outcomes in this study should be verified in human T2D patients.

## Conclusions

Currently there are no FDA-approved single agent treatments for the concurrent management of insulin sensitivity and the prevention (or at least the attenuation of progression to) to NAFLD/NASH in individuals with metabolic syndrome and T2D. The closest drug to market is obeticholic acid which recently completed a phase 3 clinical trial, but has yet to be approved by the FDA due to safety concerns in long term adverse effects [83]. Here we demonstrate that a CXCR2 antagonist, AZD5069, as a single agent, was beneficial in the prevention of progression of insulin resistance and liver pathology reminiscent of NASH/NAFLD. This single agent, or other agents in its class, may prove useful as adjunctives to T2D/metabolic syndrome standard of care. As our cellular targets of interest were neutrophils, we did not examine the effect of AZD5069 on macrophages, an important cell population involved in the effects of HFD on the progression towards liver pathology [24–35]. We expect that arresting neutrophil migration and activity will impact macrophage accumulation and function and thus, in an indirect manner, AZD5069 is expected to affect macrophages. This question is the subject of ongoing investigations in our laboratory. Our results show that AZD5069 treatment of mice on a HFD is capable of improving insulin sensitivity, decreasing hepatic lipid accumulation, and improving hepatic histopathology and circulating markers of liver function.

## Supplementary Information

**Additional file 1: Figure S1.** Representative liver sections from mice in the CN, HF, and HF + AZD diet arms NAS-scored and evaluated for markers of fibrosis (Col1a1, α-SMA, Galectin-3). A The Figure shows H&E-stained sections from mice euthanized after 16 weeks on each of the indicated diets. These sections are representative of n = 6 sections from 3 mice in each of the diet arms. The NAS score and subscores shown in Figure 5 were derived from histopathological assessments of these sections. B Immunofluorescence microscopy was conducted using primary antibodies targeting Col1a1 (5× objective), α-SMA (5× objective), and Galectin-3 (5× objective). Quantifications of immunofluorescence are shown in Figures 4c-e. The imaged field is representative of 5 randomly-selected fields among 3 separate sections from livers of 3 randomly-selected mice.

**Additional file 2: Figure S2.** Mouse liver weight and cell density. A Animal liver weight was assessed after 16 weeks on diet with marked increases in weight of mice on the HF and HF+AZD diet weight compared to CN. B Liver cell density was ascertained by DAPI staining, identifying cell nuclei. A significant decrease in cell number per field was seen in the livers of mice in the HF diet arm compared to the CN arm. Based on this, the immunofluorescence data in Figures 5 and 6 are presented as marker value per image field instead of marker value per cell number to not inflate the difference when comparisons are made to the outcomes in the HF diet arm.

## Data Availability

The datasets used and/or analysed during the current study are available from the corresponding author on reasonable request. The study agent, AZD5069, in pharmaceutical grade, can be obtained from its manufacturer, Astra Zeneca, upon direct request and materials transfer agreement. Although there may be commercial vendors for this agent, we did not test these commercially-available versions in any of our experimental work herein.

## Abbreviations

*IR*: Insulin resistance
*LP*: Liver pathology
