# Supplementary figures and images for: Improvement in insulin sensitivity and prevention of high fat diet-induced liver pathology using a CXCR2 antagonist
Source: Cardiovasc Diabetol. 2022 Jul 12;21:130. doi: 10.1186/s12933-022-01564-y (PMC9277870; doi:10.1186/s12933-022-01564-y)

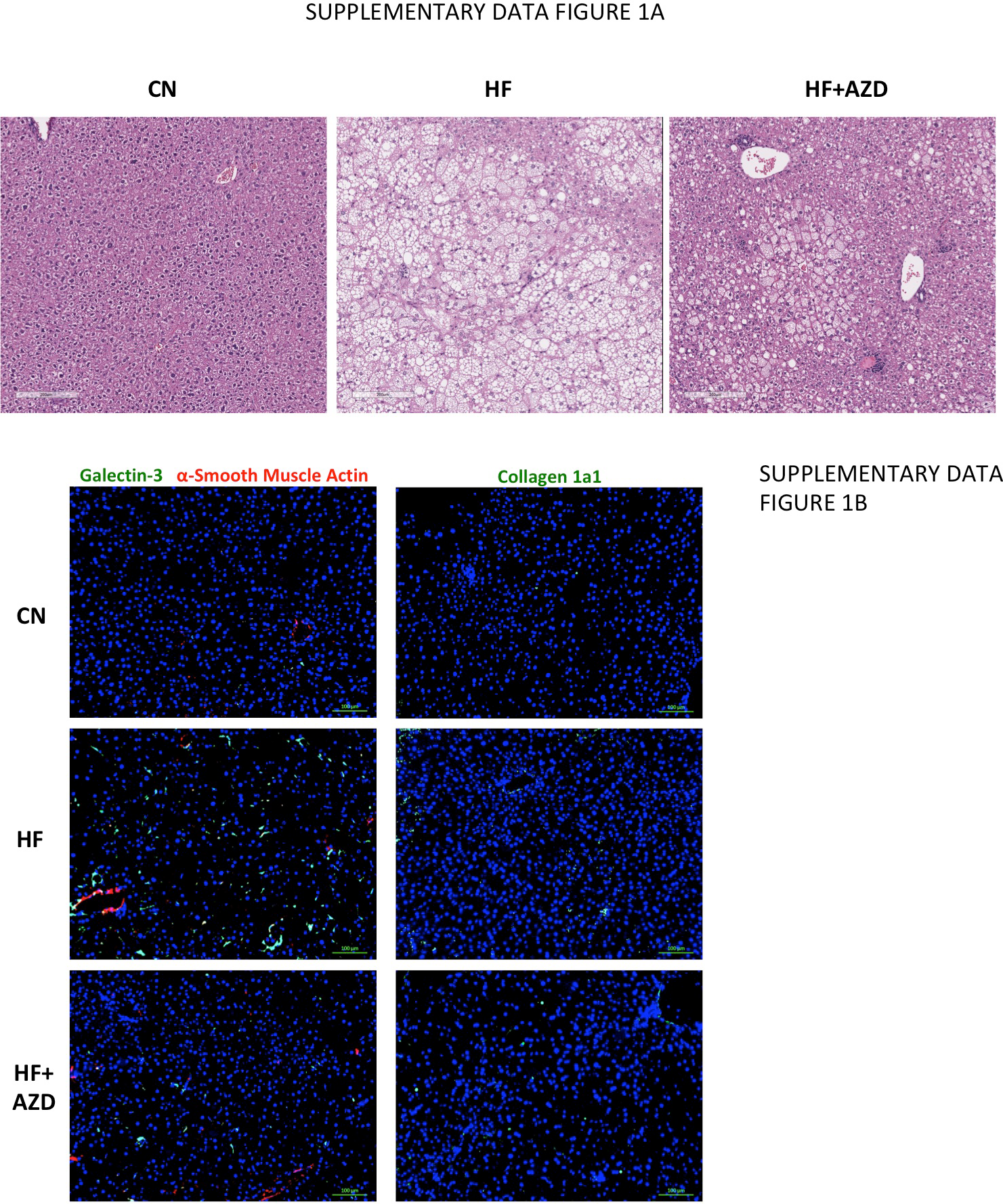

Supplement: Supplementary file 1 — Additional file 1: Figure S1. Representative liver sections from mice in the CN, HF, and HF + AZD diet arms NAS-scored and evaluated for markers of fibrosis (Col1a1, α-SMA, Galectin-3). A The Figure shows H&E-stained sections from mice euthanized after 16 weeks on each of the indicated diets. These sections are representative of n = 6 sections from 3 mice in each of the diet arms. The NAS score and subscores shown in Figure 5 were derived from histopathological assessments of these sections. B Immunofluorescence microscopy was conducted using primary antibodies targeting Col1a1 (5× objective), α-SMA (5× objective), and Galectin-3 (5× objective). Quantifications of immunofluorescence are shown in Figures 4c-e. The imaged field is representative of 5 randomly-selected fields among 3 separate sections from livers of 3 randomly-selected mice. [file 12933_2022_1564_MOESM1_ESM.tif]

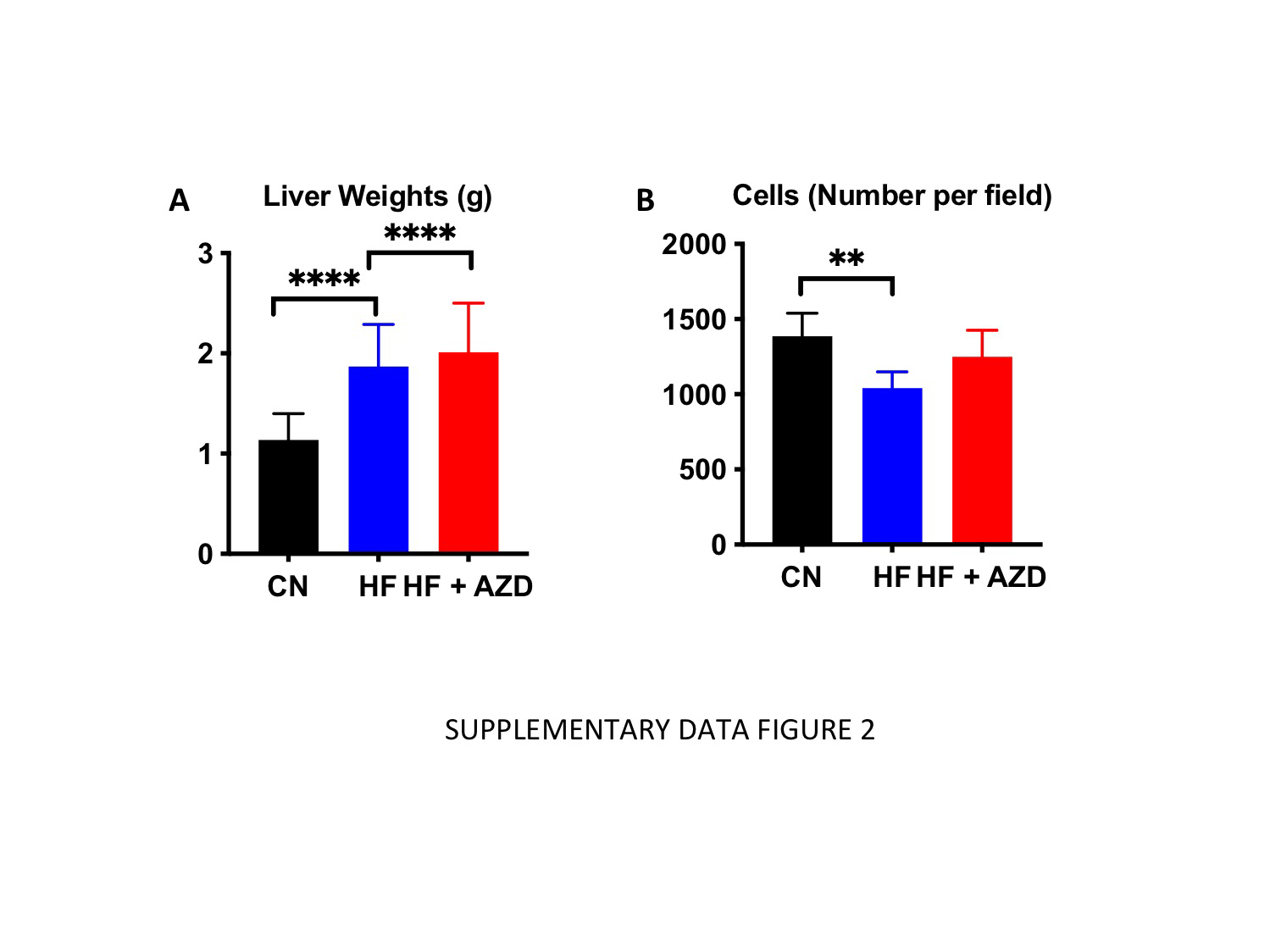

Supplement: Supplementary file 2 — Additional file 2: Figure S2. Mouse liver weight and cell density. A Animal liver weight was assessed after 16 weeks on diet with marked increases in weight of mice on the HF and HF+AZD diet weight compared to CN. B Liver cell density was ascertained by DAPI staining, identifying cell nuclei. A significant decrease in cell number per field was seen in the livers of mice in the HF diet arm compared to the CN arm. Based on this, the immunofluorescence data in Figures 5 and 6 are presented as marker value per image field instead of marker value per cell number to not inflate the difference when comparisons are made to the outcomes in the HF diet arm. [file 12933_2022_1564_MOESM2_ESM.tif]
